# Frameworks of wavelength selection in diffuse reflectance spectroscopy for tissue differentiation in orthopedic surgery

**DOI:** 10.1117/1.JBO.28.12.121207

**Published:** 2023-09-05

**Authors:** Celina L. Li, Carl J. Fisher, Katarzyna Komolibus, Konstantin Grygoryev, Huihui Lu, Ray Burke, Andrea Visentin, Stefan Andersson-Engels

**Affiliations:** a University College Cork, Biophotonics@Tyndall, IPIC, Tyndall National Institute, Cork, Ireland; b University College Cork, School of Computer Science and Information Technology, Insight Centre for Data Analytics, Cork, Ireland; c University College Cork, Department of Physics, Cork, Ireland

**Keywords:** feature selection, optimal wavelengths, ensemble learning, diffuse reflectance spectroscopy, orthopedic surgery

## Abstract

**Significance:**

Wavelength selection from a large diffuse reflectance spectroscopy (DRS) dataset enables removal of spectral multicollinearity and thus leads to improved understanding of the feature domain. Feature selection (FS) frameworks are essential to discover the optimal wavelengths for tissue differentiation in DRS-based measurements, which can facilitate the development of compact multispectral optical systems with suitable illumination wavelengths for clinical translation.

**Aim:**

The aim was to develop an FS methodology to determine wavelengths with optimal discriminative power for orthopedic applications, while providing the frameworks for adaptation to other clinical scenarios.

**Approach:**

An ensemble framework for FS was developed, validated, and compared with frameworks incorporating conventional algorithms, including principal component analysis (PCA), linear discriminant analysis (LDA), and backward interval partial least squares (biPLS).

**Results:**

Via the one-versus-rest binary classification approach, a feature subset of 10 wavelengths was selected from each framework yielding comparable balanced accuracy scores (PCA: 94.8±3.47%, LDA: 98.2±2.02%, biPLS: 95.8±3.04%, and ensemble: 95.8±3.16%) to those of using all features (100%) for cortical bone versus the rest class labels. One hundred percent balanced accuracy scores were generated for bone cement versus the rest. Different feature subsets achieving similar outcomes could be identified due to spectral multicollinearity.

**Conclusions:**

Wavelength selection frameworks provide a means to explore domain knowledge and discover important contributors to classification in spectroscopy. The ensemble framework generated a model with improved interpretability and preserved physical interpretation, which serves as the basis to determine illumination wavelengths in optical instrumentation design.

## 1 Introduction

Surgical procedures involving osteotomies pose risks of breaching into critical neurovascular and musculoskeletal structures, often leading to operative morbidity or potential mortality. One related clinical situation involves total hip and knee arthroplasty (THA/TKA) where periprosthetic fracture is considered a major complication that requires revision. Studies found that 40% and 54% of primary THA and TKA patients, respectively, encountered at least one major complication, such as fracture or one minor complication, such as neurovasculature damage, or both.^1^^,^^2^ Furthermore, with population ageing leading to rapidly growing incidence of primary arthroplasty, the number of revision surgeries will continue to increase substantially due to limited implant survivorship. The advanced surgical complexity and higher rates of associated complications have become a prevalent concern that remains to be improved. Within orthopedics, incidence projections of revision THA/TKA (rTHA/rTKA) to 2030 in the United States alone have indicated an increase of 43% to 70% and 78% to 182%, respectively, relative to the 50,000 rTHA and 72,000 rTKA cases in 2014. In particular, the patient group aged >55 years is anticipated to undergo the highest increase, suggesting that multiple revision surgeries would become necessary in the coming decades. Some common causes for rTHA include instability (28%), aseptic loosening (24%), periprosthetic fracture (18%), and prosthetic joint infection (18%).^3^

Our group previously proposed perspectives to integrating optical sensing into orthopedic surgical tools^4^ and demonstrated preliminary results for tissue differentiation based on diffuse reflectance spectroscopy (DRS) measurements of tissue types encountered in general orthopedic surgery.^5^^–^^7^ We have examined the possibility of optical integration to guide cement removal—one of the most technically challenging and time-consuming steps for surgeons during rTHA/rTKA surgery, especially removal of the distal cement plug. Choices of intraoperative image guidance modalities, such as endoscopy, fluoroscopy, or ultrasound,^8^^–^^10^ are usually surgeon dependent, which can be impractical in the operating room. Navigation through the procedure therefore relies on the surgeon’s experience and the safety measures in the surgical tool. On the other hand, extraction of well-bound bone cement can be undesirably invasive when creation of cortical windows is needed via trochanteric osteotomy to visualize the distal plug.^11^^,^^12^ Severe complications, including cortex perforation (5%) and intraoperative fracture (12%), as well as overall incomplete implant or cement extraction, can be associated with insufficient familiarity and expertise in the surgical procedure, while several techniques and instruments, such as cement-in-cement revision and ultrasonic cement removal, have been employed to ameliorate the risk.^13^^–^^15^

DRS, as a means of integrated optical guidance in complement to standard imaging modalities, has the potential to assist the orthopedic surgeon with (1) differentiating bone cement^16^ from biological tissues; (2) informing the cement-to-bone interface as a safety measure to signal pre-breaching; and (3) informing the bone-to-tissue interface as an indicator to signal post-breaching during rTHA involving cemented implants. In general, DRS allows for flexible signal processing due to the abundant information embedded in the spectral data, promoting the interest to exploit machine learning (ML) algorithms for tissue classification or physiological parameter extraction.^17^^–^^20^ However, the widely known properties of multicollinearity, redundancy, and noise affect the accuracy performance due to overfitting of the training data, as well as deteriorating interpretability and explainability of the ML model.^21^ Feature selection (FS), namely to select an optimal subset of wavelengths to the classification or regression problem, becomes crucial for understanding the knowledge space, reducing data dimensionality, and finding accurate ML models. There has been considerable research into wavelength selection in spectroscopy^22^^–^^25^ as well as generic FS algorithms,^26^^–^^29^ for which novel approaches have been proposed to process spectral data. For example, a previous study by Gunaratne et al.^30^ used multiclass Fisher’s linear discriminant analysis (LDA) to both select features and classify tissue types in ovine joint tissue specimens for orthopedic applications, achieving 100% accuracy with full DRS data of 2048 wavelengths from 190 to 1081 nm, 90% with the selected 10 wavelengths, and 70% with the selected single wavelength. Fanjul-Vélez et al.^18^ examined the use of spectral characteristics extraction and principal component analysis (PCA) as a dimensionality reduction technique on DRS measurements from *ex vivo* porcine specimens, demonstrating specificity and sensitivity values of >98%. Another study using FS by Mamouei et al.^31^ compared different variations of partial least squares (PLS) with a genetic optimization algorithm to identify important spectral regions for predicting lactate concentrations in blood using optical sensing, resulting in higher accuracy scores with a reduced number of wavelengths.

The advantage of DRS lies in the possibility of instrument miniaturization and easier integration to surgical workflows. The choice of discrete narrowband light sources in the device can be inadequate in different applications without a selection methodology. In this work, we present four distinct FS frameworks to methodologically select an optimal subset of DRS wavelengths for tissue differentiation in bone-related surgeries, achieving comparable classification accuracy using substantially fewer wavelengths. The focus is on the tailored FS frameworks to select wavelength features, which will be implemented as the light source in the optical device for reduced instrument complexity to guide bone cement removal. The primary aims are thereby defined as the following: (1) exploring domain knowledge in the orthopedics-related DRS dataset, (2) determining an optimal subset of DRS wavelengths with sufficient discriminative power between tissue types by comparing four FS frameworks, and (3) developing a suitable wavelength selection methodology for adaptation to various clinical scenarios.

## 2 Materials and Methods

All *ex vivo* measurements were approved and in compliance with the regulations at Tyndall National Institute, Cork, Ireland. “Wavelength,” namely the spectral bin width defined by the spectrometer, is referred to as “feature.” “Sample” refers to each DRS data entry. All data preprocessing and analysis were implemented in the python programming language version 3.9 (Python Software Foundation^32^) built from open-source libraries, packages, and dependencies, including scikit-learn,^33^ SciPy,^34^ and SHapley Additive exPlanations (SHAP).^35^^,^^36^

### 2.1 Specimen Preparation and DRS Data Acquisition

**Fig. 1:**
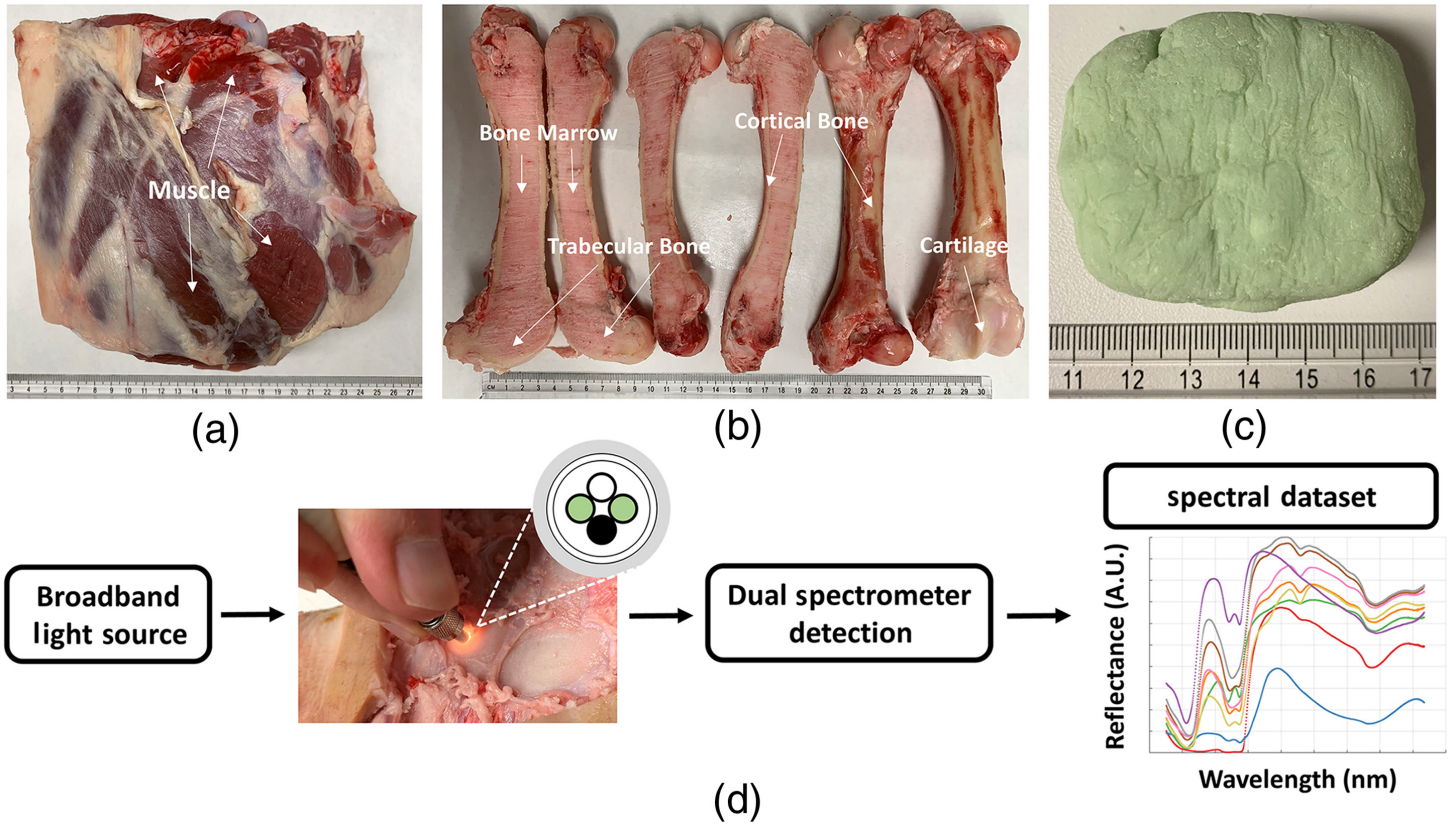
*Ex vivo* ovine tissue specimens, including (a) muscle mass; (b) femur containing bone marrow, cartilage, cortical bone, and trabecular bone; and (c) bone cement specimen. The EWDRS system in panel (d) shows the fiber optic probe, connecting to a broadband light source and two spectrometers, in contact with one tissue specimen. White circle: fiber channel to light source, green circles: fiber channels to spectrometers, and black circle: not in use.

The *ex vivo* ovine tissue specimens included bone marrow, cartilage, cortical bone, muscle, and trabecular bone sourced from a local butcher shop, which were sacrificed 2 days prior to delivery and immediately refrigerated until 2 h before measurements. All measurements were acquired at room temperature upon delivery, where tissue specimens were unprocessed and kept moist using saline spray. Muscle specimens were approximately 22×23×8  cm [Fig. 1(a)]; femoral specimens were ∼23-cm long and 3-cm in diameter with cortical bone layer of ∼5  mm [Fig. 1(b)]; and bone cement (PALACOS^®^ R+G pro, Heraeus Medical GmbH, Wehrheim, Germany) specimens were cured and hand-molded into approximately 6×5×4-cm blocks [Fig. 1(c)] 1 day earlier. DRS measurements of multiple unique locations were collected from one specimen with >5  mm apart in grid layout. The total number of tissue specimens and DRS measurements are summarized in Table 1.

**Table 1:** Total number of tissue specimens and DRS measurements used in the analysis.

	Bone marrow	Cartilage	Cortical bone	Muscle	Trabecular bone	Bone cement
Number of specimens	43	55	48	31	47	3
Number of data entries	1000	1000	1000	1000	1000	215

Figure 1(d) shows the extended-wavelength DRS (EWDRS) system, which employed a dual spectrometer detection configuration across the visible/near-infrared/short-wave infrared (VIS/NIR/SWIR) spectral range. The 355- to 1100-nm and 1100- to 1850-nm ranges were measured by QE Pro spectrometer (Ocean Insight B.V., Duiven, The Netherlands) and NIR Quest spectrometer (Ocean Insight B.V., Duiven, The Netherlands), respectively, with a tungsten-halogen broadband light source (HL-2000-HP, Ocean Insight B.V., Duiven, The Netherlands) of emission from 350 to 2400 nm. The two spectrometers were set to acquire five repeats consecutively during one acquisition of 2 s for a single measurement location. The fiber optic probe was a 1-to-4 fan-out bundle with equidistant source-detector separations of 0.63 mm (BF46LS01, 600-μm core, Low OH, Thorlabs, Munich, Germany).

### 2.2 Data Preprocessing

Each EWDRS data entry was obtained by averaging over the five repeats followed by intensity calibration and splicing the two spectra together using spline interpolation. The wavelength bin widths were ∼0.76  nm in the 355- to 1100-nm range and 1.6 nm in the 1100- to 1850-nm range per channel. All measurements were calibrated against a reflectance standard (FWS-99-01c, Avian Technologies LLC, New London, United States) and corrected for dark counts pre and post each experiment using the standard method $\frac{S_{\text{raw}}-S_{\mathrm{bkgd}}}{S_{\mathrm{ref}}-S_{\mathrm{bkgd}}}$. Savitzky–Golay (SG) data smoothing filter was applied with a frame size of 5 and polynomial order of 2 for denoising.^37^^,^^38^ The EWDRS dataset, containing numerical features and categorical labels, was structured into an m×n matrix, where m represented the number of DRS data entries and n represented the number of features. There were 5215 data entries and 1531 features subjected to further analysis. The data entries were treated as mutually independent entities.

### 2.3 Classification Models

Classification models were trained via the one-versus-rest (OVR) binary approaches. The positive class (one) was defined as bone cement, and the negative class (rest) was collectively defined for bone marrow, cartilage, cortical bone, and trabecular bone in the first scenario (boneCement versus rest). In the second scenario, the positive class was cortical bone, and the rest included bone marrow, cartilage, muscle, and trabecular bone (cortBone versus rest). Six models were included to compute balanced accuracy using all features:

• logistic regression (LogReg) representing a baseline linear regression model used for classification,
• LDA representing a baseline linear classification model,
• random forest (RF) representing an ensemble bagging classifier,
• k-nearest neighbors (KNNs) representing an instance-based classifier,
• Gaussian Naïve Bayes (GNB) representing a probabilistic classifier, and
• support vector machine (SVM) representing a maximum margin classifier.

The balanced accuracy is the arithmetic mean of sensitivity and specificity in binary classification

$$\text{Balanced accuracy}=\frac{1}{2}(\frac{\mathrm{TP}}{\mathrm{TP}+\mathrm{FN}}+\frac{\mathrm{TN}}{\mathrm{TN}+\mathrm{FP}}),$$ (1)

where TP is the true positive, TN is the true negative, FP is the false positive, and FN is the false negative. TP represented the instances of bone cement and cortical bone being correctly identified in the two scenarios, respectively. Default parameters were used. The classification model generating the highest balanced accuracy was chosen as the classifier to evaluate the quality of FS. Balanced accuracy was treated as a metric for quality check, where features of lower scores were discarded. Globally, the percentage of holdout dataset was stratified 20%. Global cross-validation (CV) was stratified 10-fold with 10 repeats. Stratified sampling was used to preserve the same class distribution of features in the training, validation, and holdout datasets as the complete dataset. The six models were trained, validated, and tested using the complete EWDRS dataset.

### 2.4 Feature Selection

**Fig. 2:**
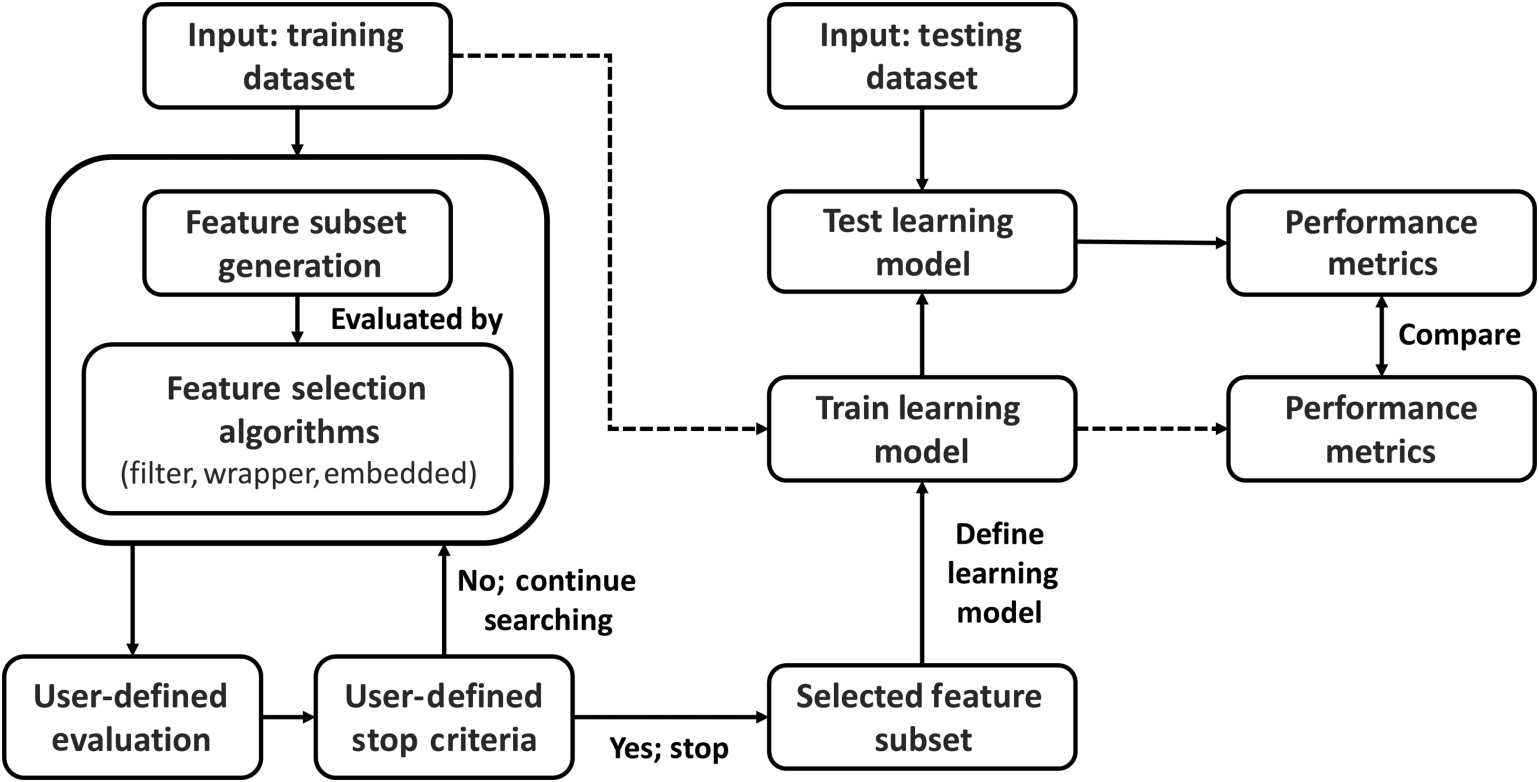
Overview of a typical FS workflow.

FS techniques were implemented via the OVR binary approach to achieve the highest discriminative power. An overview of FS process is shown in Fig. 2. Algorithms involving data transformation were incorporated with optimization techniques to permit FS using the original features. The final selection subset was pre-defined to contain 10 features. The following subsections described the implementation of PCA, LDA, and backward interval PLS (biPLS) into independent FS frameworks. Detailed explanations of the algorithms can be found in review articles.^39^^–^^42^ An ensemble framework of FS^43^ was subsequently formulated for comparison.

#### 2.4.1 Feature selection framework – principal component analysis

PCA is an unsupervised technique that projects the original data based on maximized variance onto a lower dimension via an orthogonal linear transformation. The resulted principal components (PCs) from PCA transformation, ranked by descending variance, might not bear discriminative power in the same descending order for the classification problem.^44^ Simulated annealing (SA),^45^ a stochastic optimization algorithm, was therefore implemented to search for a set of PCs that approximated the global optimum of a user-defined cost function. LDA was selected as the cost function due to its simplicity. Regularization parameters in SA were tuned by trial and error for the data domain. To minimize computational cost, the first 30 out of 1530 PCs were initially selected followed by SA optimization with 600 iterations to further select 10 PCs that jointly produced the highest LDA classification accuracy. SA optimization would be inactivated if a 100% accuracy score was generated during the first iteration. The next step involved selecting peaks from the 10 eigenvectors plotted against features based on user-defined inclusion criteria, which comprised of removal of duplicates, features within 20 nm horizontally and below 20% normalized intensity vertically. The final 10 features were determined by ranking and removing collinearity that was identifying the most relevant feature to the class label by ranked ANOVA F-values and sequentially discarding the most correlated feature to the most relevant feature based on Pearson correlation coefficients. The flowchart of PCA FS framework can be found in Fig. S1(a) in the Supplementary Material.

#### 2.4.2 Feature selection framework – linear discriminant analysis

LDA is a supervised technique that searches for a linear combination of features based on maximized class separation. To implement LDA in an FS workflow, a moving-window approach was utilized by setting a series of feature intervals of custom lengths, including 25, 50, 75, 100, 150, 200, and 300 nm. The initial feature subspace was constructed by selecting the top feature within the interval based on the ranked LDA coefficients of this interval. The computation process was iterated over all interval sizes and across the full feature domain. Duplicates and features within 30 nm of each other were discarded. The top 75% of initial feature subspace was chosen similarly by sequential removal of ranked feature collinearity, where the most relevant feature to the class label was determined by ranking LDA coefficients of the initial feature subspace. The final feature subset was calculated by performing SA optimization to search for the 10 features collectively producing the highest LDA classification accuracy. SA optimization was always activated with 2000 iterations to reach convergence. The flowchart of LDA FS framework can be found in Fig. S1(b) in the Supplementary Material.

#### 2.4.3 Feature selection framework – backward interval partial least squares

biPLS is one variation of backward variable elimination PLS methods for FS. The basis is to compare the PLS root mean square error of CV (RMSECV) of the N-1 intervals with the baseline RMSECV and eliminate the feature interval whose removal yields the lowest RMSECV in a recursive way.^46^ The baseline RMSECV was calculated using all features. Optimization of PLS components was performed for each PLS regression with 30 total components to minimize computation. RMSECV calculation was iterated over the number of intervals, the size of intervals, and the different CV shuffles of the training dataset across the full feature domain. The custom sizes of feature intervals included 20, 40, 60, 70, 80, and 95 nm. Five different CV shuffles of the training dataset were generated randomly and kept consistent. The retained feature intervals from each iteration received one vote. The features with the highest votes were deemed important. The final feature set was determined by searching the top 10 least collinear features. The most relevant feature to the class label was calculated by ranked PLS-variable importance in prediction scores.^47^ The flowchart of biPLS FS framework can be found in Fig. S2 in the Supplementary Material.

#### 2.4.4 Feature selection framework – ensemble

**Fig. 3:**
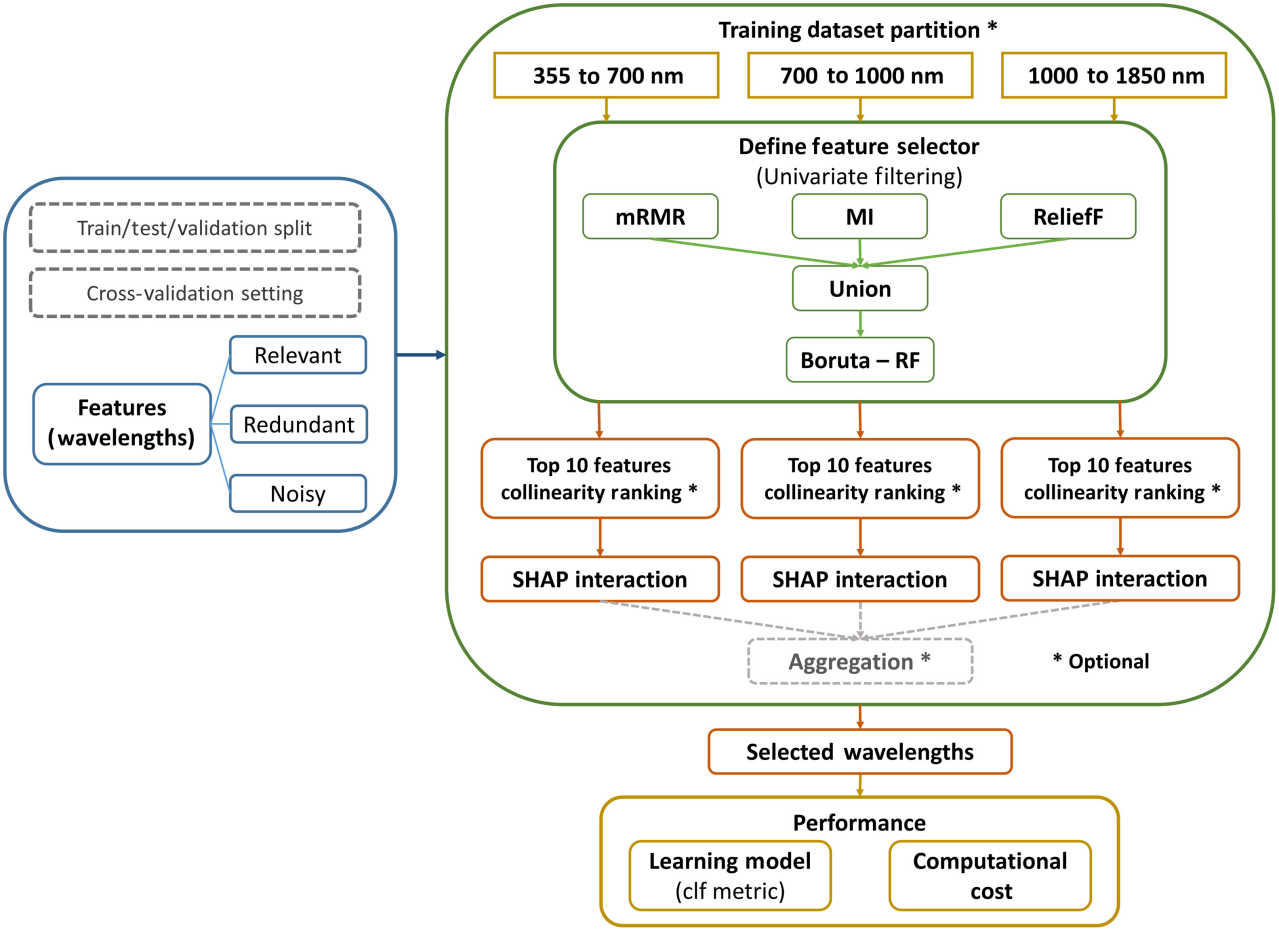
EFS framework. Optional steps are labeled with asterisks, including training dataset partition, sequential removal of collinear features by ranking, and aggregation of results from all partitions. Acronyms: mRMR for minimum redundancy – maximum relevance, MI for mutual information, Boruta-RF for Boruta random forest, SHAP for SHapley Additive exPlanations, and clf for classification.

Ensemble FS (EFS) is a learning framework that combines different FS algorithms to achieve improved outcomes by avoiding bias, merging advantages, and compensating disadvantages of individual FS methods. The EFS framework incorporated three univariate filtering methods, which were minimum redundancy – maximum relevance (mRMR)^48^ that measures the most correlation with a class; mutual information (MI)^49^ that measures the amount of information gain of one variable (feature) given the other known variable (class label); and ReliefF^50^^,^^51^ that measures conditional dependencies between the k-nearest neighbors (KNNs) of one feature in an instance-based manner. Selected features from the union of outputs by mRMR, MI, and ReliefF were input into Boruta-RF,^52^^,^^53^ which is a wrapper method around RF that utilizes shadow features to compute feature importance. The final feature subset could be determined by (1) selecting the top 10 features from Boruta-RF rank based on the ranked ANOVA F-values, resulting in a narrow spectral band (ensemble SB) or (2) selecting the top 10 least collinear features out of the Boruta-RF rank, which was computed via the same collinearity removal process in the PCA FS framework (ensemble LC). SHAP,^54^^–^^59^ one of the state of the art tools in ML explainability, was performed to explain and quantify individual contributions of selected features to model prediction base on cooperative game theory. The SHAP value was proposed by Lundberg and Lee as a unified measure to represent additive feature importance, which was the average outcomes of marginal contributions from individual features over all possible feature permutations.^36^ The SHAP algorithm in the EFS framework was based on ensembles of trees. There were two optional optimization steps, including partition of the training dataset and aggregation of the final selection. Partition involved segmenting the training dataset into multiple packets vertically by features or horizontally by samples, or both,^60^ and aggregation involved searching features from all vertical partitions to form a final subset that satisfied user-defined criteria. In the EFS framework, vertical partition by features was adopted to divide the training dataset into three spectral ranges that were approximately the VIS range of 355 to 700 nm, the NIR range of 700 to 1000 nm, and the SWIR range of 1000 to 1850 nm. Horizontal partition was indirectly performed by iterating over different compositions of CV. Aggregation was achieved by incrementally adjoining the VIS, NIR, and SWIR ranges, resulting in the VIS range of 355 to 700 nm, the VIS/NIR range of 355 to 1000, and the VIS/NIR/SWIR range of 355 to 1850 nm. The flowchart of EFS framework is shown in Fig. 3.

## 3 Results

The classification models were benchmarked on the complete dataset as a reference of the achievable maximal accuracy, followed by performance comparison of the top 1 and 10 wavelength features selected by the four FS frameworks. Further investigations examined FS in different spectral ranges and the tradeoff between the number of selected features versus model accuracy. Model explainability was explored to understand individual feature contributions to classification prediction.

### 3.1 Classification Models

The six classification models were trained with CV on the train/validation and tested on the holdout datasets containing all features [Table 2]. Two decimal places were retained for balanced accuracy. LDA was subsequently used to assess the quality of FS due to outperformance.

**Table 2:** Summary of the balanced accuracy scores on the holdout dataset generated by LogReg, LDA, RF, KNNs, GNB, and SVM and the average computation time to train one model.

Classifier	Balanced accuracy (%)		Computation time (s)
	BoneCement versus rest	CortBone versus rest	
LogReg	100	95.68 ± 1.38	0.25
LDA	100	99.79 ± 0.33	1.4
RF	99.98 ± 0.05	97.70 ± 1.14	2.0
KNN	100	96.37 ± 1.32	0.21
GNB	99.98 ± 0.05	87.49 ± 1.75	0.12
SVM	100	95.48 ± 1.34	0.64

### 3.2 PCA, LDA, and biPLS Feature Selection Frameworks

**Fig. 4:**
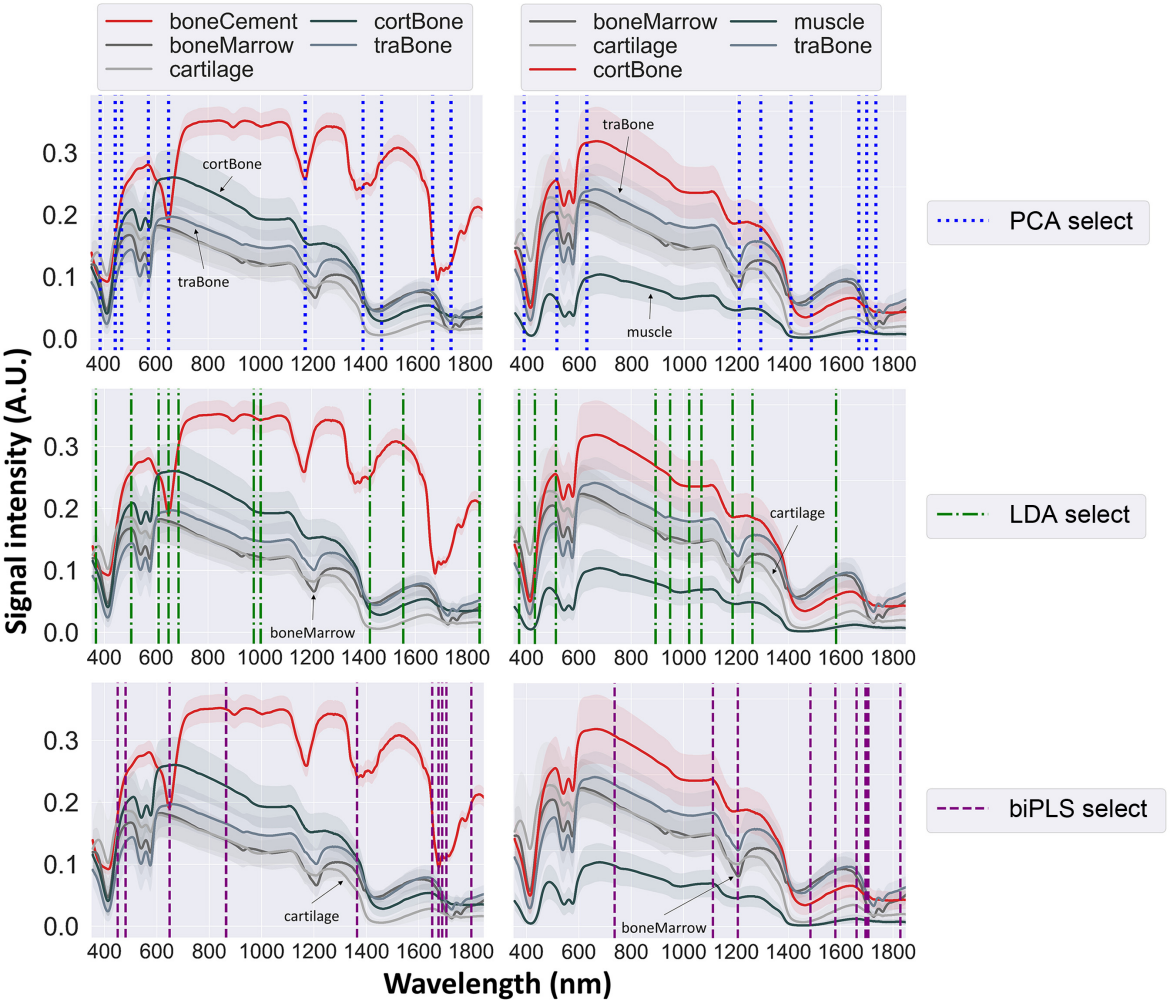
The final 10 selected features from FS frameworks of PCA (top), LDA (middle), and biPLS (bottom) on the EWDRS spectra for boneCement versus rest (left) and cortBone versus rest (right). Standard deviations are shown in shaded error bands. The arrows highlight spectra of different class labels in subfigures for each scenario. Acronyms: boneCement for bone cement, boneMarrow for bone marrow, cortBone for cortical bone, traBone for trabecular bone, PCA for principal component analysis, LDA for linear discriminant analysis, and biPLS for backward interval partial least squares.

The final 10 selected wavelength features from FS frameworks of PCA, LDA, and biPLS overlaid on the EWDRS spectra for boneCement versus rest and cortBone versus rest are shown in Fig. 4. Balanced accuracy scores are shown in Table 3 (top) with the most relevant features to classification and the average computation time to execute one framework.

**Table 3:** Summary of the balanced accuracy scores using the top 1 and 10 wavelength features generated from PCA, LDA, biPLS, and EFS frameworks for boneCement versus rest and cortBone versus rest, the averaged computation time for one entire framework (top), and the scores generated by EFS framework on the holdout dataset in the VIS and VIS/NIR ranges (bottom). Accuracy using the top 1 wavelength was not applicable for ensemble SB.

(Top)		BoneCement versus rest					CortBone versus rest				
FS framework balanced accuracy (%)		PCA	LDA	biPLS	Ensemble VIS/NIR/SWIR		PCA	LDA	biPLS	Ensemble VIS/NIR/SWIR	
					SB	LC				SB	LC
Top 1 wavelength	Train/ validation	100	100	99.98 ± 0.05	100	100	88.06 ± 2.15	84.98 ± 2.23	88.09 ± 2.15	N/A	88.13 ± 2.16
	Holdout	100	100	100	100	100	87.31 ± 3.91	83.69 ± 4.81	87.36 ± 3.90	N/A	87.36 ± 3.89
	Top feature (nm)	1463	1424	1801	1457	1458	1211	1188	1207	1208	1209
Top 10 wavelengths	Train/ validation	100	100	100	100	100	96.21 ± 1.39	98.41 ± 0.82	96.12 ± 1.39	95.24 ± 1.48	96.56 ± 1.27
	Holdout	100	100	100	100	100	94.78 ± 3.47	98.16 ± 2.02	95.80 ± 3.04	93.74 ± 3.24	95.77 ± 3.16
Computation time (min)		0.64	0.16	9072	4.6	4.6	21	1.1	10656	4.6	4.6
**(Bottom)**		**BoneCement versus rest**				**CortBone versus rest**					
**FS framework balanced accuracy (%)**		**Ensemble VIS/NIR**		**Ensemble VIS**		**Ensemble VIS/NIR**		**Ensemble VIS**			
		**SB**	**LC**	**SB**	**LC**	**SB**	**LC**	**SB**	**LC**		
Holdout		99.94 ± 0.19	100	98.99 ± 0.77	100	92.07 ± 3.44	93.38 ± 3.36%	86.84 ± 3.90	91.95 ± 4.09		
Top feature (nm)		990	956	555	576	925	927	695	699		

### 3.3 Ensemble Framework of Feature Selection

**Fig. 5:**
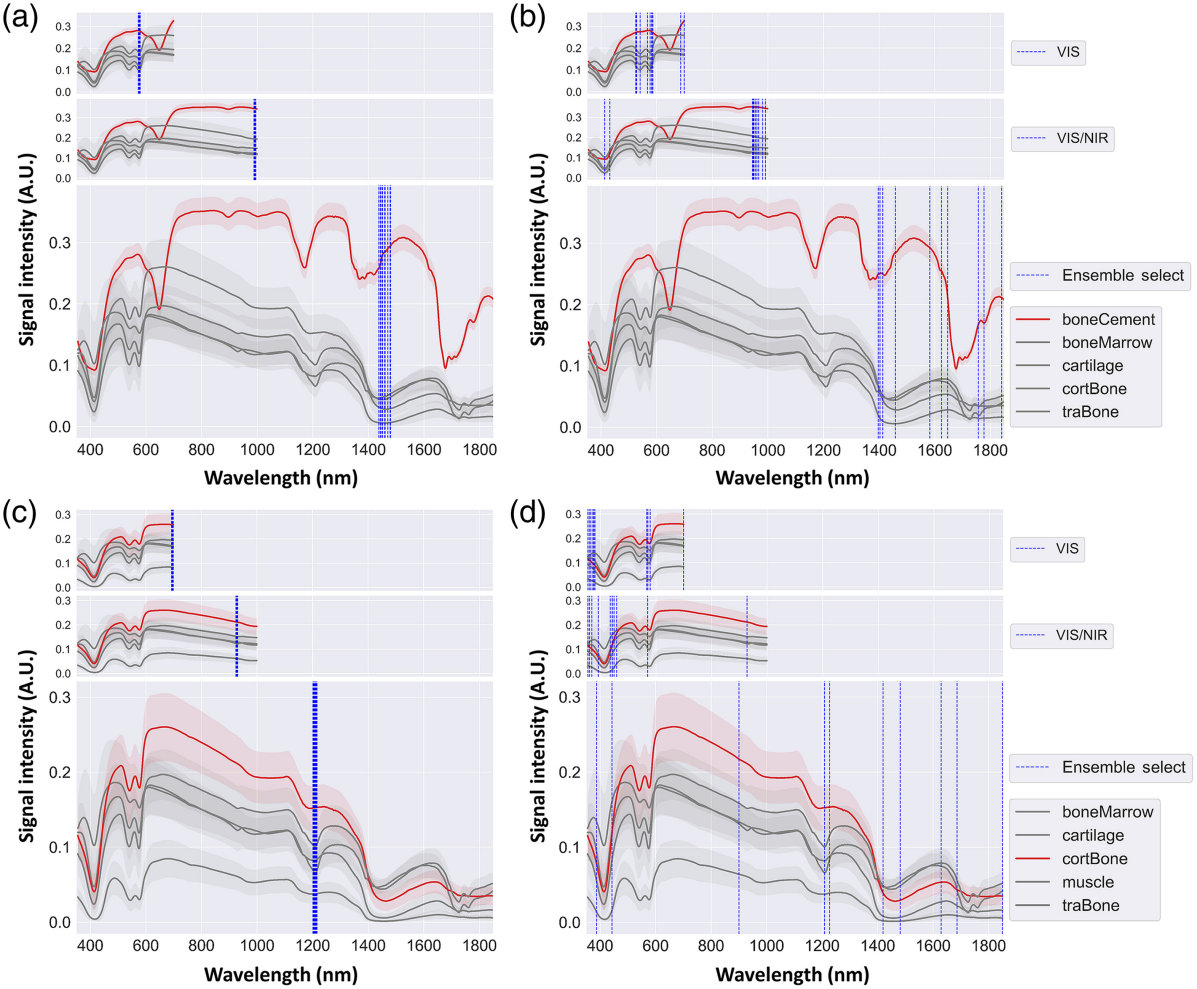
The final 10 selected wavelength features from ensemble SB as spectral band (a,c) and ensemble LC as the least collinear features (b,d) for boneCement versus rest (a,b) and cortBone versus rest (c,d) in the VIS (top), VIS/NIR (middle), and VIS/NIR/SWIR (bottom) spectral ranges, respectively, on the EWDRS spectra. Standard deviations are shown in shaded error bands. Acronyms: boneCement for bone cement, boneMarrow for bone marrow, cortBone for cortical bone, and traBone for trabecular bone.

The final 10 wavelength features overlaid on the EWDRS spectra in the VIS, VIS/NIR, and VIS/NIR/SWIR ranges are shown in Fig. 5 with balanced accuracy scores in Table 3 (top) and (bottom). Optimal performance was discovered using ensemble LC and biPLS FS frameworks based on the high averaged accuracy over the two holdout sets for the top 1 and 10 features. The latter framework, however, required excessive computational effort. The final subset generated by ensemble SB collectively yielded a narrow spectral band, making its deployment comparable to using the top feature.

**Fig. 6:**
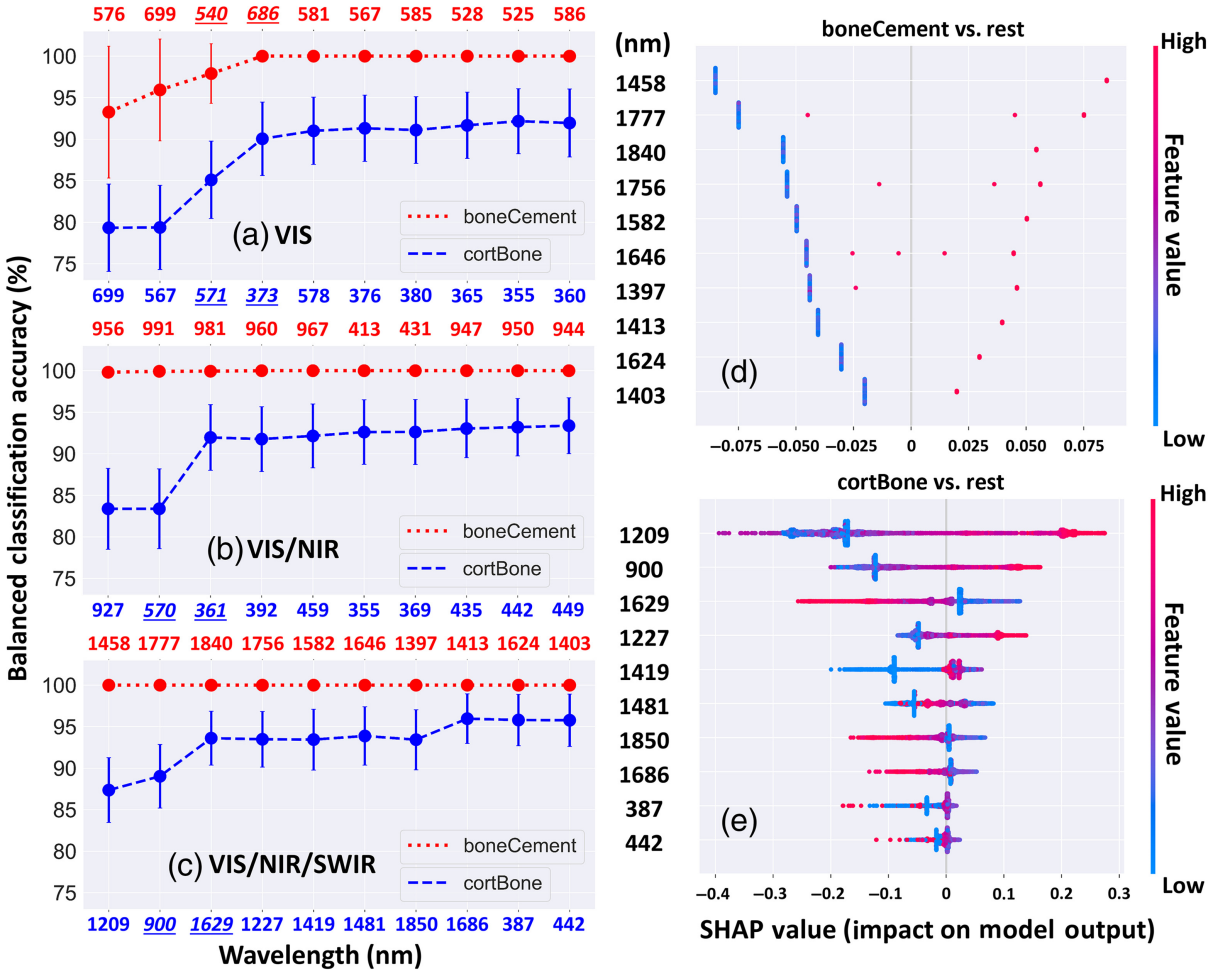
Balanced accuracy computed by LDA with CV plotted against sequential inclusion of the ranked least collinear wavelength features for boneCement versus rest (top x-axis) and cortBone versus rest (bottom x-axis) in panels (a) VIS, (b) VIS/NIR, and (c) VIS/NIR/SWIR ranges. The underscored features indicated the two wavelengths approaching the first plateau. Distribution of individual feature contributions on the prediction by SHAP analysis is shown for (d) boneCement versus rest and (e) cortBone versus rest. The 10 features were ranked on the vertical axis in descending order of their contributions to prediction. The horizontal axis illustrates the impact level of individual features of high or low value related to positive or negative prediction. Acronyms: LDA for linear discriminant analysis, CV for cross-validation, and SHAP for SHapley Additive exPlanations.

Figures 6(a)–6(c) demonstrated the top 10 least collinear features on the x-axis ranked by contribution to classification for boneCement versus rest (top) and cortBone versus rest (bottom), as well as the balanced accuracy calculated using sequential inclusion of the ranked wavelength features. The accuracy scores reached the first plateau after ∼3 to 4 wavelength features. Distribution of individual feature contributions on the prediction by SHAP analysis is shown in Figs. 6(d) and 6(e), where the vertical axis illustrated the 10 features ranked in descending order of contribution and the SHAP value on the horizontal axis explained the association between the individual feature values and the level of their impact on the target prediction. The SHAP plot visualized contributions from all instances determined by SHAP values on each feature row, which included 4215 and 5000 data points for boneCement versus rest and cortBone versus rest, respectively. The red and blue indicated high and low feature values, which corresponded to high and low DRS signal intensity, respectively. Positive and negative SHAP values represented positive and negative contribution by the exact amount in log odds as additive feature importance to the binary model output, respectively. The 1458- and 1209-nm features had the strongest effect on the prediction of positive classes in the two scenarios. For boneCement versus rest, the model prediction was more likely to be bone cement when the DRS signal intensity increased for all the 10 wavelength features. For cortBone versus rest, a positive correlation was illustrated at wavelength features 1209 and 900 nm whilst wavelengths 1629 and 1850 nm displayed an inverse correlation with DRS signal intensity.

## 4 Discussion

The EWDRS dataset, created by measuring DRS in ovine specimens of tissue types commonly encountered in orthopedics-related surgery, was explored to achieve the primary aims. In this study, we implemented PCA, LDA, and PLS into discovery-orientated FS frameworks and constructed an EFS framework for comparison to understand domain knowledge and determine an optimal subset of wavelengths with high discriminative power for the positive class. The feature inclusion criterion of 20- or 30-nm separation enforced selection of unique features by matching the full width at half maximum (FWHM) of an average light-emitting diode (LED), which was further refined by sequential ranking and removal of high correlation. The moving-window approach also alleviated the effect of multicollinearity by separating wavelength features using interval partitioning and manual deletions of adjacent features. By applying multicollinearity reduction, the final selected wavelengths were sufficiently distinguishable by LED source(s) of certain limited bandwidths. On the other hand, multicollinearity offers the advantage of selecting multiple different feature subsets with similar classification outcomes, enabling flexibility in hardware design. This behavior was demonstrated by clusters of the selected features from all four FS frameworks. The disadvantage is also evident that it deteriorates model interpretability especially in models involving data transformation due to lost physical representations and less inferential model coefficients. The wavelengths of light sources selected by transformed coefficients became less reliable in practice. The EFS framework comprising of three univariate filters and one tree model was created to resolve the issue. The 10 wavelength features by the EFS framework offered comparable balanced accuracy to using all features, which could be reduced to 3 to 4 wavelength features. An optimized FS framework for subsequent deployment should be implemented with feature engineering to merge collinear features into one new feature, therefore decreasing computational burden and redundant outcomes. The tradeoff between the number of wavelength features and classification accuracy should also be considered to optimize instrument complexity and cost. Other conventional methods, such as first and second derivatives, could also provide decisive information for FS in spectroscopy.

The four FS frameworks considered correlations among the final 10 selected wavelengths. In general, prominent absorption peaks of biomarkers were identified, such as collagen, lipid, and water in the SWIR range as well as different forms of hemoglobin (Hb) in the VIS/NIR range. For both scenarios, absorption regions for lipids at 1210 nm, collagen at 1200, 1500, and 1725 nm, and water at 1440 nm were recognized with some contribution from Hb at 576 nm.^61^ The lack of tissue chromophores in the non-biological specimens contributed to the selection of wavelength features in the spectral region with stronger absorption from biological specimens by amplifying the difference in signal intensity. Furthermore, FS frameworks implementing PCA, LDA, and biPLS systematically selected one absorption peak of lipid as the most relevant features in cortBone versus rest, including 1211, 1188, and 1207 nm of <25  nm apart and statistically insignificant difference due to limited resolution of the DRS system [Table 3(top)]. The fine resolution calculated by FS frameworks might not be precisely characterized by hardware because of the limited FWHM of an average LED. Larger FWHM widths could be preferred over laser diodes of narrower bandwidths. By encompassing more wavelengths, such as the band in ensemble SB covering 1200 to 1216 nm [Fig. 5(c)], the balanced accuracy was improved from 88% to 94%. For boneCement versus rest, PCA and LDA FS frameworks both selected wavelengths at around 1460 nm corresponding to one absorption peak of water. The biPLS FS framework chose the top wavelength in the farther SWIR region at 1800 nm with no selection around 1460 in the final subset. Balanced accuracy was nevertheless comparable. Such observations demonstrated multicollinearity and implied drawbacks of utilizing core algorithms based on data transformation. The initial pool of all-relevant features generated by the algorithms might not translate to high classification scores in the original data space even with optimization techniques. Second, collinear features were removed from the pool by discarding adjacent wavelengths of similar discriminative strength, leading to a final subset that contained relevant features of minimal predictive power. Wavelength selection in Fig. 4 included relevant features corresponding to non-specific absorbers in biological tissues presumably due to the difference in signal intensity in the original data space. Features selected by the LDA FS framework covered the 800 to 1000 nm range, over which prominent tissue chromophores demonstrate broader absorption spectra. Moreover, LDA was used in both classification and FS, contributing to the highest balanced accuracy for the 10-wavelength classification with improved model performance in Table 3 (top). The final subset of wavelength features tended to fall in the SWIR region of 1100 to 1800 nm, especially for boneCement versus rest, with some selection from the VIS region of 400 to 600 nm [Figs. 4 and 5], suggesting that the higher level of signal intensity in addition to the absorption signatures contributed greater predictive power than absorption features alone. During orthopedic surgery with less active bleeding, this behavior demonstrated utility of the SWIR region as the illumination wavelengths over which Hb exhibited minimal absorption and offered advantages to reducing the effect of blood contamination on DRS measurements.

In a similar vein, the final subset from ensemble LC included relevant features with minimal predictive power, rendering the order of predictive features within the rank crucial. In Figs. 6(a)–6(c), the balanced accuracy curves reached the first plateau with inclusion of the first 3 to 4 features though the increase was statistically insignificant in Fig. 6(c). For cortBone versus rest, the highest balanced accuracy was reached in the VIS/NIR/SWIR range. Increase of the classification score was observed as more features in the NIR and SWIR ranges were added stepwise for evaluation. A comparable pattern was shown for boneCement versus rest with a final 100% balanced accuracy individually achieved in the three spectral ranges. The least collinear features however formed clusters at certain wavelengths in the VIS and VIS/NIR ranges for both scenarios [Figs. 5(b) and 5(d)], suggesting that these spectral ranges contained fewer distinct features of high discriminative power. The EFS framework likewise selected spectral bands at maximized signal intensity inside the VIS (top) and VIS/NIR (middle) ranges for both scenarios in Figs. 5(a) and 5(c). Contribution of Hb absorption at around 550 nm was only deemed important with multicollinearity removal as seen in the VIS and VIS/NIR ranges by comparing ensemble LC with ensemble SB. In the literature, Gunaratne et al.^30^ concluded that wavelengths with the most discriminative power largely existed in the spectral range of 370 to 470 nm and 800 to 1010 nm where Hb, water, and lipid could be major contributors to classification, corresponding to the findings in our study.

Limitations of this work stemmed from dataset creation, which was designed for data mining and measured in a static laboratory environment with two detection fibers measuring different acquisition volumes. The exact results might not be generalizable to other situations, such as an ongoing surgery or a different experimental setup generating new DRS datasets from other species of different ages. Under such circumstances, rigorous data preprocessing and experimental setup will be required to standardize and normalize across different datasets. New features or class labels will also need to be engineered to address external environmental factors. It is nevertheless believed that the FS frameworks can be adapted to new scenarios of changed class labels, given that assumptions of the algorithms are met. For future directions, wavelengths selected from the EFS framework, including 1210 and 1460 nm, will be validated in *ex vivo* studies and subsequently implemented in the optical probe as the light sources for further translational experiments in *in vivo* subjects. An ML system with continual learning will be established using one-class classification to identify bone cement. The initial train/validation dataset of bone cement in various conditions will be collected including ageing, blood contamination, and hydration at body temperature.

## 5 Conclusions

The present work described four different FS frameworks to select important wavelength features for tissue differentiation in orthopedics. Three FS frameworks were constructed by implementing conventionally used algorithms, including PCA, LDA, and PLS, while the other was formulated via an ensemble approach. All frameworks generated comparable results and model performance. The EFS framework produced more interpretable models with efficiency. The final subset of 10 selected features contained wavelengths corresponding to prominent absorption peaks of major tissue chromophores, such as Hb, water, lipid, and collagen at around 600, 1200, 1400, and 1500 nm, respectively, and wavelengths at maximized signal intensity difference. FS results set the groundwork to choose adequate light source(s) in the optical device for bone cement removal guidance in rTHA surgery. In the future, the frameworks will be adapted to various clinical applications to facilitate the determination of important wavelengths and biomarker sensitivity.

## Code, Data, and Materials Availability

The EWDRS dataset used for the analysis is available in a public Github repository or Zenodo repository (DOI: 10.5281/zenodo.7554759) all licensed under CC-BY-NC-SA-4.0:

https://github.com/Biophotonics-Tyndall/PUB-FeatureSelectionDataset.git.

The code in.ipynb format used to generate the results, and figures are available in a public Github repository or Zenodo repository (DOI: 10.5281/zenodo.7554778) all licensed under CC-BY-NC-SA-4.0:

https://github.com/Biophotonics-Tyndall/PUB-FeatureSelectionCode.git.

Supplementary Material can be found in a public Github licensed under CC-BY-NC-SA-4.0:

https://github.com/Biophotonics-Tyndall/PUB-FeatureSelectionSuppl.git.

The authors intend to submit a complementary data descriptor article to scientific data subsequently upon acceptance of this article.

## References

[1] Kagan R. et al., “Complications and pitfalls of direct anterior approach total hip arthroplasty,” Ann. Jt. 3(5), 1–7 (2018). 10.21037/aoj.2018.04.05

[2] Heo S. M. et al., “Complications to 6 months following total hip or knee arthroplasty: observations from an Australian clinical outcomes registry,” BMC Musculoskelet. Disord. 21(602), 1–11 (2020). 10.1186/s12891-020-03612-8 PMC7488141 32912197

[3] Schwartz A. M. et al., “Projections and epidemiology of revision hip and knee arthroplasty in the United States to 2030,” J. Arthroplast. 35(6), S79–S85 (2020). 10.1016/j.arth.2020.02.030 PMC7239745 32151524

[4] Fisher C. et al., “Perspective on the integration of optical sensing into orthopedic surgical devices,” J. Biomed. Opt. 27(1), 010601 (2022). JBOPFO 1083-3668 10.1117/1.JBO.27.1.010601 34984863 PMC8727454

[5] Li C. L. et al., “Wavelength selection using diffuse reflectance spectra and machine learning algorithms for tissue differentiation in orthopedic surgery,” in Biophotonics Congr.: Biomed. Opt. 2022 (Transl. Microsc., OCT, OTS, BRAIN), p. TS4B.6 (2022). 10.1364/translational.2022.ts4b.6

[r6] Grygoryev K. et al., “Cranial perforation using an optically-enhanced surgical drill,” IEEE Trans Biomed Eng 67(12), 3474–3482 (2020). 10.1109/TBME.2020.2987952 32310759

[7] Duperron M. et al., “Diffuse reflectance spectroscopy-enhanced drill for bone boundary detection,” Biomed. Opt. Express 10(2), 961 (2019). BOEICL 2156-7085 10.1364/BOE.10.000961 30800526 PMC6377869

[8] Govaers K. et al., “Endoscopy for cement removal in revision arthroplasty of the hip,” J. Bone Jt. Surg. 88-A(Suppl. 4), 101–110 (2006). JBJSB4 0021-9355 10.2106/JBJS.F.00699 17142440

[r9] Slotkin E. M. Patel P. D. Suarez J. C., “Accuracy of fluoroscopic guided acetabular component positioning during direct anterior total hip arthroplasty,” J. Arthroplast. 30(9, Suppl. 1), 102–106 (2015). 10.1016/j.arth.2015.03.046 26105615

[10] Sdao S. et al., “The role of ultrasonography in the assessment of peri-prosthetic hip complications,” J. Ultrasound 18(3), 245–250 (2015). 10.1007/s40477-014-0107-4 26261466 PMC4529409

[11] Laffosse J. M., “Removal of well-fixed fixed femoral stems,” Orthop. Traumatol. Surg. Res. 102(1), S177–S187 (2016). 10.1016/j.otsr.2015.06.029 26797009

[12] Masri B. A. Mitchell P. A. Duncan C. P., “Removal of solidly fixed implants during revision hip and knee arthroplasty,” J. Am. Acad. Orthop. Surg. 13(1), 18–27 (2005). 10.5435/00124635-200501000-00004 15712979

[13] Tovar-Bazaga M. et al., “Surgical technique of a cement-on-cement removal system for hip and knee arthroplasty revision surgery,” Arthroplast. Today 9, 112–117 (2021). 10.1016/j.artd.2021.05.008 34189215 PMC8217307

[r14] Cnudde P. H. J. et al., “Cement-in-cement revision of the femoral stem,” Bone Jt. J. 99-B(4), 27–32 (2017). 10.1302/0301-620X.99B4.BJJ-2016-1222.R1 28363891

[15] Liddle A. et al., “Ultrasonic cement removal in cement-in-cement revision total hip arthroplasty: what is the effect on the final cement-in-cement bond?” Bone Jt. Res. 8(6), 246–252 (2019). 10.1302/2046-3758.86.BJR-2018-0313.R1 PMC6609863 31346452

[16] Vaishya R. Chauhan M. Vaish A., “Bone cement,” J. Clin. Orthop. Trauma 4(4), 157–163 (2013). 10.1016/j.jcot.2013.11.005 26403875 PMC3880950

[17] Engelhardt A. et al., “Comparing classification methods for diffuse reflectance spectra to improve tissue specific laser surgery,” BMC Med. Res. Methodol. 14(1), 1–15 (2014). 10.1186/1471-2288-14-91 25030085 PMC4136948

[r18] Fanjul-Vélez F. Pampín-Suárez S. Arce-Diego J. L., “Application of classification algorithms to diffuse reflectance spectroscopy measurements for ex vivo characterization of biological tissues,” Entropy 22(7), 736 (2020). ENTRFG 1099-4300 10.3390/e22070736 33286511 PMC7517275

[r19] Dahlstrand U. et al., “Extended-wavelength diffuse reflectance spectroscopy with a machine-learning method for in vivo tissue classification,” PLoS One 14(10), e0223682 (2019). POLNCL 1932-6203 10.1371/journal.pone.0223682 31600296 PMC6786558

[20] Nguyen M. H. et al., “Machine learning to extract physiological parameters from multispectral diffuse reflectance spectroscopy,” J. Biomed. Opt. 26(5), 052912 (2021). JBOPFO 1083-3668 10.1117/1.JBO.26.5.052912

[21] Chan J. Y. et al., “Mitigating the multicollinearity problem and its machine learning approach: a review,” Mathematics 10(8), 1283 (2022). 10.3390/math10081283

[22] Silalahi D. D. et al., “Robust wavelength selection using filter-wrapper method and input scaling on near infrared spectral data,” Sensors 20(17), 5001 (2020). SNSRES 0746-9462 10.3390/s20175001 32899292 PMC7506801

[r23] Kaneko H. et al., “Transfer learning and wavelength selection method in NIR spectroscopy to predict glucose and lactate concentrations in culturemedia using VIP-Boruta,” Anal. Sci. Adv. 2(9–10), 470–479 (2021). 10.1002/ansa.202000177 PMC10989590 38716444

[r24] Baltussen E. J. M. et al., “Optimizing algorithm development for tissue classification in colorectal cancer based on diffuse reflectance spectra,” Biomed. Opt. Express 10(12), 6096–6113 (2019). BOEICL 2156-7085 10.1364/BOE.10.006096 31853388 PMC6913395

[25] Jiang H. Xu W. Chen Q., “Comparison of algorithms for wavelength variables selection from near-infrared (NIR) spectra for quantitative monitoring of yeast (Saccharomyces cerevisiae) cultivations,” Spectrochim. Acta - Part A Mol. Biomol. Spectrosc. 214(5), 366–371 (2019). 10.1016/j.saa.2019.02.038 30802792

[26] Remeseiro B. Bolon-canedo V., “A review of feature selection methods in medical applications,” Comput. Biol. Med. 112, 103375 (2019). CBMDAW 0010-4825 10.1016/j.compbiomed.2019.103375 31382212

[r27] Jović A. Brkić K. Bogunović N., “A review of feature selection methods with applications,” in 38th Int. Convention on Inf. and Commun. Technol., Electron. and Microelectron. (MIPRO), pp. 1200–1205 (2015). 10.1109/MIPRO.2015.7160458

[r28] Peng Y. Wu Z. Jiang J., “A novel feature selection approach for biomedical data classification,” J. Biomed. Inf. 43(1), 15–23 (2010). 10.1016/j.jbi.2009.07.008 19647098

[29] Bolón-Canedo V. Sánchez-Maroño N. Alonso-Betanzos A., “A review of feature selection methods on synthetic data,” Knowl. Inf. Syst. 34, 483–519 (2013). 10.1007/s10115-012-0487-8

[30] Gunaratne R. et al., “Wavelength weightings in machine learning for ovine joint tissue differentiation using diffuse reflectance spectroscopy (DRS),” Biomed. Opt. Express 11(9), 5122 (2020). BOEICL 2156-7085 10.1364/BOE.397593 33014603 PMC7510883

[31] Mamouei M. et al., “Comparison of wavelength selection methods for in-vitro estimation of lactate: a new unconstrained, genetic algorithm-based wavelength selection,” Sci. Rep. 10(1), 16905 (2020). SRCEC3 2045-2322 10.1038/s41598-020-73406-4 33037265 PMC7547666

[32] Python Software Foundation, Python Programming Language. Version 3.9, https://www.python.org/.

[33] Pedregosa F. et al., “Scikit-learn: machine learning in Python,” J. Mach. Learn. Res. 12, 2825–2830 (2011).

[34] Virtanen P. et al., “SciPy 1.0: fundamental algorithms for scientific computing in Python,” Nat. Methods 17, 261–272 (2020).1548-7091 10.1038/s41592-019-0686-2 32015543 PMC7056644

[35] Lundberg S. M. et al., “From local explanations to global understanding with explainable AI for trees,” Nat. Mach. Intell. 2(1), 56–67 (2020). 10.1038/s42256-019-0138-9 32607472 PMC7326367

[36] Lundberg S. M. Lee S.-I., “A unified approach to interpreting model predictions,” in 31st Conf. Neural Inf. Process. Syst. (NIPS 2017) (Section 2), pp. 4766–4775 (2017).

[37] Krishnan S. R. Seelamantula C. S., “On the selection of optimum Savitzky-Golay filters,” IEEE Transactions on Signal Processing 61(2), 380–391 (2013). ITPRED 1053-587X 10.1109/TSP.2012.2225055

[38] Rinnan Å. Van den Berg F. Engelsen S. B., “Review of the most common pre-processing techniques for near-infrared spectra,” Trends Anal. Chem. 28(10), 1201–1222 (2009). 10.1016/j.trac.2009.07.007

[39] Abdi H. Williams L. J., “Principal component analysis,” Wiley Interdiscip. Rev. Comput. Stat. 2(4), 433–459 (2010). 10.1002/wics.101

[r40] Tharwat A. et al., “Linear discriminant analysis: a detailed tutorial,” AI Commun. 30(2), 169–190 (2017). ACMMEE 0921-7126 10.3233/AIC-170729

[r41] Mehmood T. et al., “A review of variable selection methods in Partial Least Squares Regression,” Chemom. Intell. Lab. Syst. 118, 62–69 (2012). 10.1016/j.chemolab.2012.07.010

[42] Lee L. C. Liong C. Y. Jemain A. A., “Partial least squares-discriminant analysis (PLS-DA) for classification of high-dimensional (HD) data: a review of contemporary practice strategies and knowledge gaps,” Analyst 143(15), 3526–3539 (2018). ANLYAG 0365-4885 10.1039/C8AN00599K 29947623

[43] Bolón-Canedo V. Alonso-Betanzos A., “Ensembles for feature selection: a review and future trends,” Inf. Fusion 52, 1–12 (2019). 10.1016/j.inffus.2018.11.008

[44] Pechenizkiy M. Tsymbal A. Puuronen S., “PCA-based feature transformation for classification: issues in medical diagnostics,” in Proc. 17th IEEE Symp. Comput. Med. Syst., Vol. 17, pp. 535–540 (2004).

[45] Bertsimas D. Tsitsiklis J., “Simulated annealing,” Stat. Sci. 8(1), 10–15 (1993). STSCEP 0883-4237 10.1214/ss/1177011077

[46] Leardl R. Norgaard L., “Sequential application of backward interval partial least squares and genetic algorithms for the selection of relevant spectral regions,” J. Chemom. 18(11), 486–497 (2004). JOCHEU 0886-9383 10.1002/cem.893

[47] Chong I. G. Jun C. H., “Performance of some variable selection methods when multicollinearity is present,” Chemom. Intell. Lab. Syst. 78(1), 103–112 (2005). 10.1016/j.chemolab.2004.12.011

[48] Ding C. Peng H., “Minimum redundancy feature selection from microarray gene expression data,” J. Bioinf. Comput. Biol. 3(2), 185–205 (2005).0219-7200 10.1142/S0219720005001004 15852500

[49] Vergara J. R. Estévez P. A., “A review of feature selection methods based on mutual information,” Neural Comput. Appl. 24(1), 175–186 (2014). 10.1007/s00521-013-1368-0

[50] Robnik-Šikonja M. Kononenko I., “Theoretical and empirical analysis of relieff and rrelieff,” Mach. Learn. 53, 23–69 (2003). MALEEZ 0885-6125 10.1023/A:1025667309714

[51] Urbanowicz R. J. et al., “Relief-based feature selection: introduction and review,” J. Biomed. Inf. 85, 189–203 (2018). 10.1016/j.jbi.2018.07.014 PMC6299836 30031057

[52] Kursa M. B. Jankowski A. Rudnicki W. R., “Boruta - a system for feature selection,” Fundam. Inf. 101(4), 271–285 (2010). 10.3233/FI-2010-288

[53] Kursa M. B. Rudnicki W. R., “Feature selection with the Boruta package,” J. Stat. Software 36(11), 1–13 (2010). 10.18637/jss.v036.i11

[54] Ribeiro M. T. Singh S. Guestrin C., “‘Why should I trust you?’ Explaining the predictions of any classifier,” in Proc. 22nd ACM SIGKDD Int. Conf. Knowl. Discov. and Data Mining, pp. 97–101 (2016).

[r55] Štrumbelj E. Kononenko I., “Explaining prediction models and individual predictions with feature contributions,” Knowl. Inf. Syst. 41(3), 647–665 (2014). 10.1007/s10115-013-0679-x

[r56] Shrikumar A. Greenside P. Kundaje A., “Learning important features through propagating activation differences,” in 34th Int. Conf. Mach. Learn., ICML 2017, Vol. 7, pp. 4844–4866 (2017).

[r57] Datta A. Sen S. Zick Y., “Algorithmic transparency via quantitative input influence: theory and experiments with learning systems,” in Proc. - 2016 IEEE Symp. Secur. and Privacy, SP 2016, pp. 598–617 (2016). 10.1109/SP.2016.42

[r58] Bach S. et al., “On pixel-wise explanations for non-linear classifier decisions by layer-wise relevance propagation,” PLoS One 10(7), e0130140 (2015). POLNCL 1932-6203 10.1371/journal.pone.0130140 26161953 PMC4498753

[59] Lipovetsky S. Conklin M., “Analysis of regression in game theory approach,” Appl. Stoch. Model. Bus. Ind. 17(4), 319–330 (2001). 10.1002/asmb.446

[60] Morán-Fernández L. Bolón-Canedo V. Alonso-Betanzos A., “Centralized vs. distributed feature selection methods based on data complexity measures,” Knowl.-Based Syst. 117, 27–45 (2017). 10.1016/j.knosys.2016.09.022

[61] Wilson R. H. et al., “Review of short-wave infrared spectroscopy and imaging methods for biological tissue characterization,” J. Biomed. Opt. 20(3), 030901 (2015). JBOPFO 1083-3668 10.1117/1.JBO.20.3.030901 25803186 PMC4370890

