# Supplementary material for: Frameworks of wavelength selection in diffuse reflectance spectroscopy for tissue differentiation in orthopedic surgery
Source: J Biomed Opt. 2023 Sep 5;28(12):121207. doi: 10.1117/1.JBO.28.12.121207 (PMC10479945; doi:10.1117/1.JBO.28.12.121207)
Supplement: Supplementary file 1 [file JBO_028_121207_SD001.pdf]

Supplementary information

Principal Component Analysis and Linear Discriminant Analysis Feature Selection Frameworks

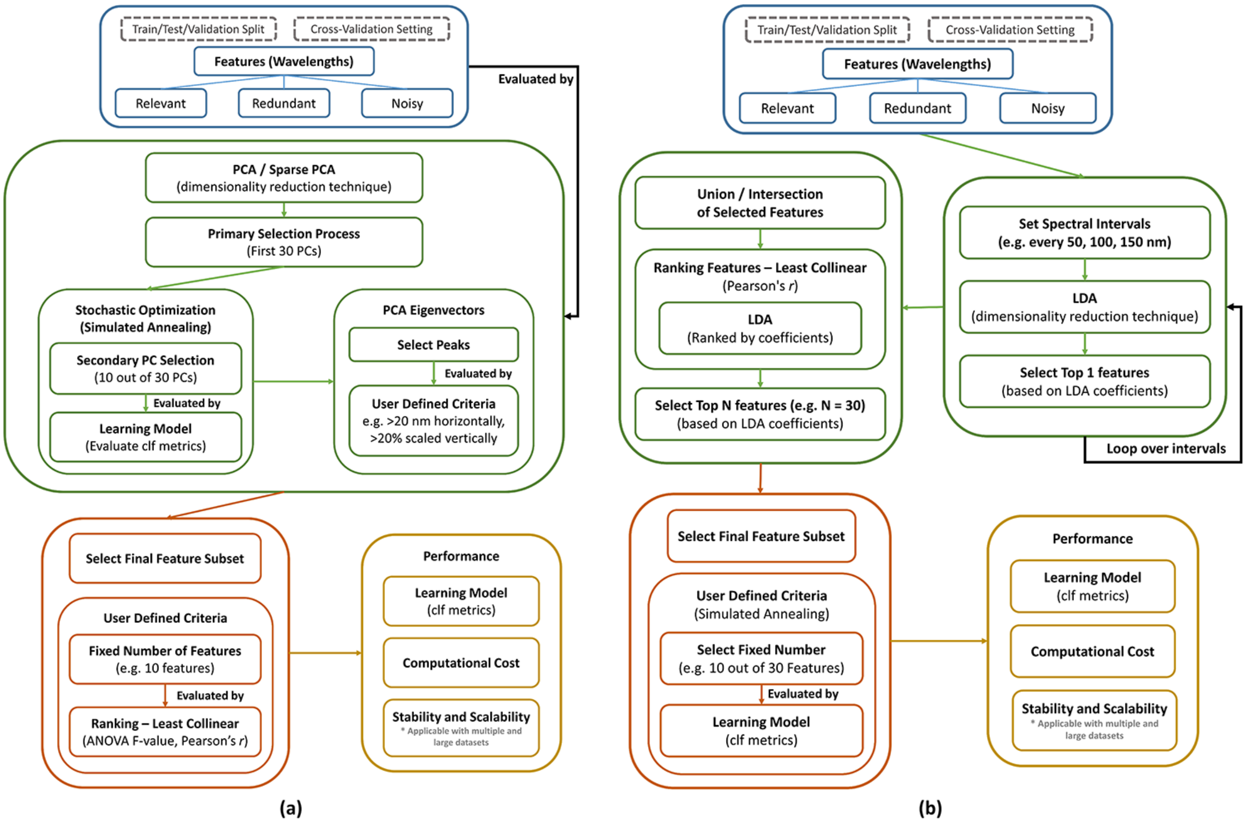

Figure S1. FS framework implementing (a) PCA/Sparse PCA and (b) LDA to process the EWDRS dataset. Acronyms: FS for feature selection; PCA for principal component analysis; PC for principal component; LDA for linear discriminant analysis; EWDRS for extended-wavelength diffuse reflectance spectroscopy.

Backward Interval Partial Least Squares Feature Selection Framework

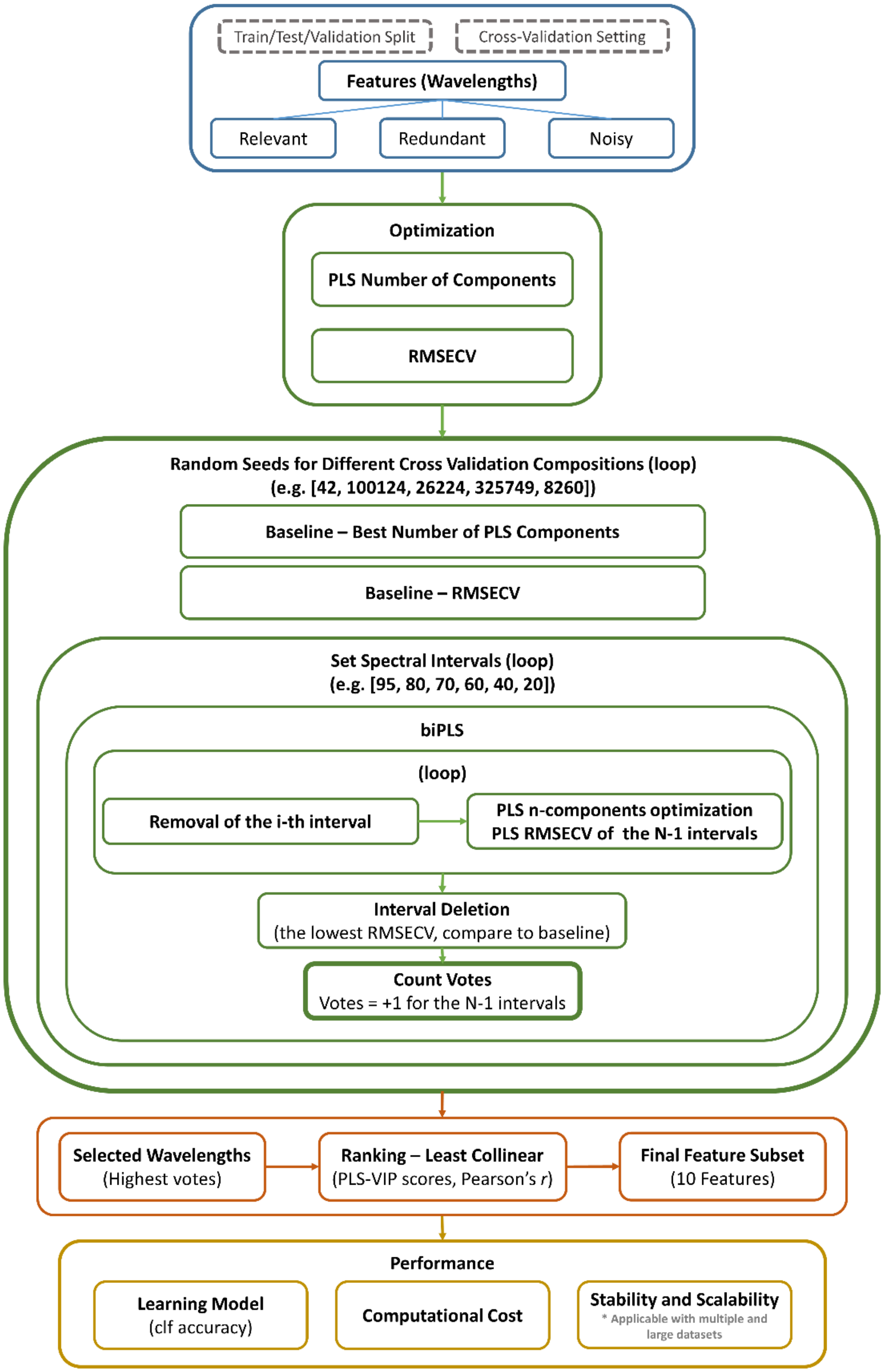

Figure S2. biPLS FS framework showing the workflow to process EWDRS dataset. Acronyms: biPLS for backward interval partial least squares; FS for feature selection; EWDRS for extended-wavelength diffuse reflectance spectroscopy; PLS for partial least squares; PLS-VIP for PLS-Variable Importance in Prediction; RMSECV for root mean square error of cross-validation; clf for classification.
